# Assessment of cerebral hemodynamic changes in acute ischemic stroke patients following mechanical thrombectomy using CT perfusion imaging

**DOI:** 10.3389/fneur.2025.1628717

**Published:** 2025-09-25

**Authors:** Chao Tian, Jing Chen, Junfeng Zhang, Feizhou Du

**Affiliations:** ^1^Department of Radiology, The General Hospital of Western Theater Command, Chengdu, China; ^2^Department of Radiology, Sichuan Modern Hospital, Chengdu, China

**Keywords:** acute ischemic stroke, mechanical thrombectomy, CT perfusion, cerebral hemodynamics, recanalization, clinical outcomes

## Abstract

**Background:**

Acute ischemic stroke (AIS) is a leading cause of disability and death in China, with mechanical thrombectomy (MT) being an effective treatment for AIS due to large vessel occlusion (LVO). The aim of this study was to evaluate the efficacy of MT in AIS patients using CT perfusion (CTP) imaging to assess cerebral hemodynamics before and after the procedure.

**Methods:**

This retrospective study enrolled 76 AIS patients with unilateral anterior circulation LVO (internal carotid artery or middle cerebral artery M1/M2 segments) who underwent MT at The General Hospital of Western Theater Command PLA from January 2020 to April 2023. All patients underwent pre- and post-thrombectomy CTP, with the interval between scans ≤72 h. Successful recanalization was defined as achieving modified Thrombolysis in Cerebral Infarction (mTICI) grade ≥2b (reperfusion of ≥50% of the ischemic territory) on immediate post-thrombectomy angiography. Primary outcomes included changes in perfusion abnormality range and CTP parameters [relative cerebral blood flow (rCBF), relative cerebral blood volume (rCBV), relative mean transit time (rMTT), relative time to peak (rTTP), relative time to maximum of the residual function (rTmax)]. Secondary outcomes included 7-day NIHSS score improvement and 90-day modified Rankin Scale (mRS) scores.

**Results:**

Post-thrombectomy, 69 patients (90.8%) achieved successful recanalization (mTICI ≥ 2b), including 44 (57.9%) with mTICI 3 and 25 (32.9%) with mTICI 2b. CTP within 3 days post-MT showed significant increases in rCBF (0.560 ± 0.11 vs. 1.020 ± 0.29, *P* < 0.01) and rCBV (0.850 ± 0.13 vs. 1.010 ± 0.15, *P* < 0.01), and decreases in rMTT (1.41 vs. 1.03, *P* < 0.01), rTTP (1.380 ± 0.12 vs. 1.050 ± 0.12, *P* < 0.01), and rTmax (3.71 ± 0.1 vs. 1.40 ± 0.9, *P* < 0.01) compared to baseline. Perfusion abnormalities resolved in 36 patients (47.4%), reduced in 30 (39.5%), and showed no improvement in 10 (13.2%). Patients with resolved perfusion defects had higher rates of 7-day NIHSS improvement [89.7% vs. 60.7% (reduced) and 30.0% (no improvement), *P* = 0.001 and *P* = 0.011] and 90-day good outcomes (mRS 0–2: 83.3% vs. 33.3% and 20.0%, both *P* < 0.01). Hyperperfusion occurred in 15 (21.7%) successfully recanalized patients, with 73.3% achieving good 90-day outcomes.

**Conclusion:**

CTP imaging is a valuable tool for assessing MT efficacy in AIS patients. Post-thrombectomy CTP detects significant improvements in cerebral hemodynamics, with resolved perfusion defects strongly predicting favorable clinical outcomes. These findings support the utility of CTP in monitoring post-treatment recovery and guiding clinical decision-making.

## 1 Introduction

Acute ischemic stroke (AIS), often precipitated by arterial stenosis or occlusion leading to cerebral tissue ischemia and hypoxia, represents a prevalent cerebrovascular condition in clinical practice (1, 2). Globally, ischemic stroke accounts for ~80% of all stroke cases, while in China, this proportion is even higher, with AIS comprising ~87% of strokes. The incidence of AIS remains stubbornly high in China, exacerbated by an aging population and shifts in lifestyle, making it the most frequent type of stroke in the country (3). The primary goal of AIS treatment is to improve cerebral blood circulation, with revascularization therapies, including intravenous thrombolysis and mechanical thrombectomy, being the most effective during the acute phase (4). Among these, mechanical thrombectomy (MT) has emerged as a cornerstone intervention for large vessel occlusion (LVO)-related AIS, significantly improving functional outcomes compared to intravenous thrombolysis alone. However, incomplete recanalization (achieving mTICI 0–2a) still occurs in 10%−20% of cases, and even among successfully recanalized patients (mTICI ≥ 2b), those with mTICI 2c or 3 (complete or near-complete reperfusion) exhibit superior 90-day functional independence rates compared to those with mTICI 2b (5, 6).

Intravenous thrombolysis, typically facilitated by alteplase, is the first-line approach for revascularization. However, clinical experiences have revealed suboptimal recanalization rates following alteplase administration, which correlates with poorer outcomes (7). Consequently, MT has become the primary focus for LVO-AIS, with research emphasizing optimization of recanalization efficacy, reduction of post-procedural complications, and enhancement of long-term prognosis.

CT perfusion (CTP) imaging is a powerful tool for quantifying and reflecting changes in local tissue blood flow, with numerous studies utilizing it to assess cerebral hemodynamic alterations in patients with ischemic cerebrovascular diseases (8). Post-MT CTP imaging holds critical clinical value: it enables detection of residual ischemia, evaluation of tissue reperfusion adequacy, prediction of hemorrhagic transformation and functional outcomes, and guidance for individualized post-procedural management (9, 10). Yet, there is a paucity of literature exploring the relationship between CTP parameters and clinical outcomes in patients with anterior circulation AIS following intravenous thrombolysis. CTP imaging allows for rapid, non-invasive evaluation of cerebral hemodynamics, and with advancements in imaging technology, it now offers whole-brain coverage, enabling precise assessment of abnormal perfusion areas in brain tissue (11). Stroke is the second leading cause of disability and death globally and the primary cause in China, with AIS accounting for ~87% of cases. Large vessel occlusion is one of the main etiologies of AIS (12). Mechanical thrombectomy, an effective treatment for AIS due to large vessel occlusion, can restore blood supply to ischemic tissue, salvage the ischemic penumbra, and improve patient prognosis (13). Previous research has indicated that CTP-derived hemodynamic parameters can reflect changes in cerebral hemodynamics before and after thrombolysis in AIS patients (14).

In this study, we utilized CTP imaging to assess cerebral hemodynamic changes before and after MT in patients with anterior circulation LVO strokes, aiming to evaluate the utility of CTP in assessing the efficacy of thrombectomy.

## 2 Patients and methods

### 2.1 Patients

**Figure 1:**
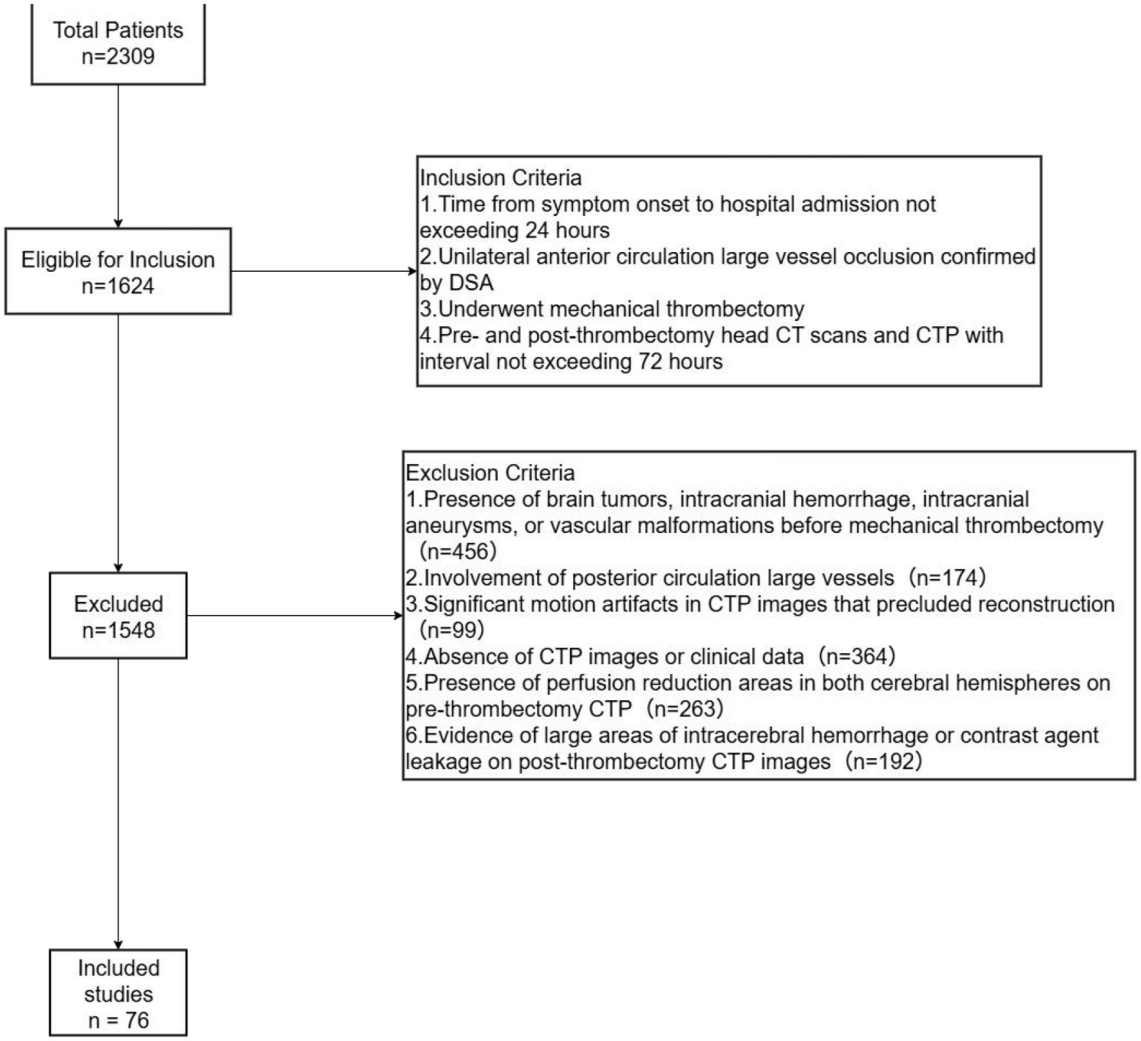
Inclusion and exclusion flow chart.

This retrospective study consecutively enrolled patients with acute ischemic stroke (AIS) who underwent mechanical thrombectomy at The General Hospital of Western Theater Command PLA through the Acute Stroke Green Channel from January 2020 to April 2023. The diagnosis of AIS adhered to the criteria outlined in the “Chinese Guidelines for the Diagnosis and Treatment of Acute Ischemic Stroke 2018” (15). Patient selection for mechanical thrombectomy followed the screening standards set forth in the “Chinese Guidelines for Endovascular Treatment of Acute Ischemic Stroke 2018” (15). Inclusion criteria were as follows: (1) time from symptom onset to hospital admission not exceeding 24 h; (2) unilateral anterior circulation large vessel occlusion (internal carotid artery or middle cerebral artery M1, M2 segments) confirmed by DSA; (3) underwent mechanical thrombectomy; (4) both pre- and post-thrombectomy, patients underwent head CT scans and CTP, with the interval between CTP scans not exceeding 72 h. Exclusion criteria included: (1) presence of brain tumors, intracranial hemorrhage, intracranial aneurysms, or vascular malformations before mechanical thrombectomy; (2) involvement of posterior circulation large vessels; (3) significant motion artifacts in CTP images that precluded reconstruction; (4) absence of CTP images or clinical data; (5) presence of perfusion reduction areas in both cerebral hemispheres on pre-thrombectomy CTP; (6) evidence of large areas of intracerebral hemorrhage or contrast agent leakage on post-thrombectomy CTP images. The study protocol was reviewed and approved by the Ethics Committee of The General Hospital of Western Theater Command PLA (Ethics NO.2022EC2-ky043). The process of enrolling patients in this study is shown in Figure 1.

### 2.2 Methods

#### 2.2.1 Data collection

Demographic data were collected, including gender, age, time from last known well to admission, baseline National Institutes of Health Stroke Scale (NIHSS) scores, and admission Alberta Stroke Program Early CT Score (ASPECTS) (16). Surgical data were also collected, including the site of vascular occlusion (internal carotid artery and middle cerebral artery M1, M2 segments), preoperative intravenous thrombolysis, postoperative vascular recanalization, and postoperative patient clinical outcomes.

#### 2.2.2 Mechanical thrombectomy procedure and evaluation of vascular recanalization

All mechanical thrombectomy treatments were performed by a neurointerventional team with over 5 years of surgical experience. The Seldinger technique was used to puncture the femoral artery and place an 8-F sheath. After angiographic confirmation of the occlusion site, thrombectomy techniques (including aspiration thrombectomy, stent thrombectomy, or combined techniques) were determined. Immediate post-thrombectomy angiography assessed the modified Thrombolysis in Cerebral Infarction (mTICI) grade, with grades ≥2b considered successful recanalization (10). If successful recanalization was achieved, the procedure was terminated. If recanalization was not achieved after more than five attempts, the procedure was concluded, and conservative medical treatment was chosen.

#### 2.2.3 CT scan and CTP examination methods

All patients underwent head CT scans and CTP using a 256-slice spiral CT scanner (Model Revolution; GE Healthcare, USA) before and within 3 days after mechanical thrombectomy. Routine head CT scans were performed with a slice thickness and interval of 5 mm. Subsequently, whole-brain CTP was conducted with a tube voltage of 70 kV and a tube current of 100 mAs. All patients were administered 40 ml of iodinated contrast medium (Iopromide, 370 mgI/ml; Bayer Healthcare, Germany) via a power injector through the antecubital or forearm vein, followed immediately by 40 ml of isotonic saline, both injected at a rate of 6 ml/s. Scanning began 4 s after injection and lasted for 60 s.

#### 2.2.4 CTP image analysis method

A CTP intelligent analysis system (Version 2.4.3; Shukun Technology Co., Ltd.) was used to analyze pre- and post-thrombectomy CTP raw images. The healthy side's middle cerebral artery was set as the input artery, and the superior sagittal sinus as the output vein. The system automatically calculated a series of perfusion parameter pseudo-color images, including cerebral blood flow (CBF), cerebral blood volume (CBV), mean transit time (MTT), time to peak (TTP), and time to maximum of the residual function (Tmax). The CTP intelligent analysis system presented different values of perfusion parameters in pseudo-color images, allowing for the identification of perfusion abnormalities on CTP images. Perfusion abnormalities were characterized by reduced CBF in ischemic areas, with or without reduced CBV, and delayed MTT, TTP, and Tmax. Two neuroimaging physicians with over 5 years of experience independently analyzed the pre- and post-treatment perfusion pseudo-color images. The largest perfusion abnormality area on the Tmax image before treatment was measured, and the region of interest was manually outlined along the delayed area on the affected side compared to the healthy side, with the healthy side's region of interest generated automatically by mirroring. The CBF, CBV, MTT, TTP, and Tmax values of the corresponding regions on both the affected and healthy sides were measured, and the values on the affected side were divided by those on the healthy side to obtain relative perfusion parameter values [i.e., relative cerebral blood flow (rCBF), relative cerebral blood volume (rCBV), relative mean transit time (rMTT), relative time to peak (rTTP), and relative time to maximum of the residual function (rTmax)]. The system automatically calculated the volume of the infarct core (rCBF < 30% area) and the perfusion reduction area (Tmax > 6 s area). Post-treatment perfusion parameter measurements were taken on the same slice as the pre-treatment images, with regions of interest outlined to match the pre-treatment shape and size. The same method was used to obtain post-treatment relative perfusion parameter values, and hyperperfusion on the post-treatment CTP images was defined as the affected side showing higher CBF and CBV than the healthy side, with a lower Tmax. The measurements of the two physicians were averaged to determine the final results, and in cases of significant differences, the two physicians consulted to reach a consensus.

#### 2.2.5 Grouping and clinical outcome assessment

Patients were categorized based on the change in perfusion abnormality range on post-thrombectomy CTP images compared to pre-thrombectomy images into three groups: no improvement (increased or no significant change in perfusion abnormality range), reduced perfusion abnormality range, and disappeared perfusion abnormality range.

NIHSS scores were used to evaluate neurological changes at 7 days post-thrombectomy or at discharge. Following previous research, an improvement in NIHSS scores was defined as a ΔNIHSS score (difference between baseline NIHSS score and post-treatment NIHSS score) >4 or a post-treatment NIHSS score of 0–1. Clinical outcomes were assessed at 90 days post-treatment using the modified Rankin Scale (mRS), with scores of 0–2 indicating good outcomes and 3–6 indicating poor outcomes.

### 2.3 Statistical analysis

Relative perfusion parameter values that were normally distributed before and after mechanical thrombectomy were expressed as mean ± standard deviation and analyzed using paired sample *t*-tests. Those not normally distributed were expressed as median and interquartile range [M (P25, P75)] and analyzed using the Wilcoxon rank-sum test. Quantitative data that were normally distributed were expressed as mean ± standard deviation, and those not normally distributed were expressed as median and interquartile range [M (P25, P75)]. Comparisons among the three groups were made using one-way ANOVA for data that were normally distributed and had equal variances; otherwise, the Kruskal–Wallis test was used. Categorical data were expressed as numbers and percentages (%), and comparisons among the three groups were made using chi-square tests or Fisher's exact probability method. A *P*-value < 0.05 was considered statistically significant. Data analysis was performed using SPSS 25.0 statistical software.

## 3 Results

### 3.1 General information

A total of 76 AIS patients were included, consisting of 45 males and 31 females, with ages ranging from 34 to 97 years and an average age of 65 ± 12 years. The time from last known normal to hospital admission varied from 0 to 19 h, with a median time of 6 (2, 10) h. The baseline NIHSS scores ranged from 4 to 26, with a median score of 13 (10, 19). Twenty-eight patients underwent intravenous thrombolysis followed by mechanical thrombectomy, while 48 patients underwent mechanical thrombectomy alone. Angiography during the procedure confirmed the occlusion sites to be the internal carotid artery in 20 cases, the M1 segment of the middle cerebral artery in 47 cases, and the M2 segment in 9 cases.

Immediate post-thrombectomy, 69 patients (90.8%) achieved successful recanalization, with 44 (57.9%) achieving mTICI grade 3 and 25 (32.9%) achieving mTICI grade 2b. Five patients (6.5%) did not achieve successful recanalization, with 2 (2.6%) having mTICI grade 2a and 3 (3.9%) having mTICI grade 0 (Table 1).

**Table 1:** Baseline characteristics and treatment details of AIS patients with successful and unsuccessful recanalization.

**Characteristics**	**Total (*n* = 76)**	**Successful Recanalization (*n* = 69)**	**Unsuccessful Recanalization (*n* = 7)**	***t*/χ2/*Z***	***P*-value**
**Demographics**					
Male, *n* (%)	45 (59.2)	41 (59.4)	4 (57.1)	\(χ^2^ = 0.015\)	0.912
Age, mean ± SD (years)	65 ± 12	65 ± 12	64 ± 14	0.182	0.857
**Clinical presentation**					
Time from last known normal to admission, median (IQR) (h)	6 (2, 10)	6 (2, 10)	7 (3, 11)	0.487	0.632
Baseline NIHSS score, median (IQR)	13 (10, 19)	12 (10, 18)	18 (15, 22)	2.326	0.021
Hypertension, *n* (%)	49 (64.5)	44 (63.8)	5 (71.4)	0.128	0.726
Diabetes mellitus, *n* (%)	28 (36.8)	25 (36.2)	3 (42.9)	0.102	0.765
Atrial fibrillation, *n* (%)	23 (30.3)	20 (29.0)	3 (42.9)	0.563	0.451
Smoking history, *n* (%)	31 (40.8)	28 (40.6)	3 (42.9)	0.013	0.903
**Treatment details**					
Intravenous thrombolysis before MT, *n* (%)	28 (36.8)	25 (36.2)	3 (42.9)	0.102	0.765
**Imaging features**					
Occlusion site, *n* (%)				0.637	0.517
Internal carotid artery	20 (26.3)	18 (26.1)	2 (28.6)	–	–
MCA M1 segment	47 (61.8)	43 (62.3)	4 (57.1)	–	–
MCA M2 segment	9 (11.8)	8 (11.6)	1 (14.3)	–	–
Admission ASPECTS, median (IQR)	8 (7, 9)	8 (7, 9)	7 (6, 8)	2.031	0.043
Collateral circulation (MP-CTA score ≥2), *n* (%)	51 (67.1)	49 (71.0)	2 (28.6)	5.582	0.018
**CT perfusion parameters (Pre-MT)**					
Infarct core volume, median (IQR) (ml)	8.0 (2.2, 32.0)	7.5 (2.0, 30.0)	15.0 (5.5, 45.0)	2.124	0.037
Perfusion deficit volume, mean ± SD (ml)	149.4 ± 71.1	145 ± 68	178 ± 82	2.015	0.049
**Time parameters**					
Time from symptom onset to imaging, median (IQR) (h)	2.5 (1.0, 4.0)	2.3 (1.0, 3.8)	3.5 (2.0, 5.0)	1.824	0.068
Time from symptom onset to treatment, median (IQR) (h)	5.0 (3.0, 8.0)	4.8 (2.8, 7.5)	7.0 (5.5, 9.0)	2.017	0.044

### 3.2 CT scans

The initial head CT scans upon admission for the 76 patients showed ASPECTS scores of 8 (7, 9), with 67 cases (88.2%) exhibiting mild reductions in brain tissue density and blurred gray-white matter boundaries, indicative of early ischemic signs. Postoperative head CT scans revealed low-density infarction areas in all 76 patients.

### 3.3 Comparison of CTP

Pre-emergency CTP scans for all 76 patients revealed perfusion abnormalities consistent with clinical manifestations, characterized by reduced CBF in ischemic regions, with or without reduced CBV, and delays in MTT, TTP, and Tmax. The infarct core volume was 8.0 (2.2, 32.0) ml, and the perfusion reduction area volume was (149 ± 71) ml. These perfusion abnormalities were predominantly localized to the territories of the occluded arteries: 20 cases (26.3%) in the internal carotid artery (ICA) territory, 47 cases (61.8%) in the middle cerebral artery (MCA) M1 segment territory, and 9 cases (11.8%) in the MCA M2 segment territory, corresponding to the confirmed occlusion sites.

CTP scans within 3 days post-thrombectomy showed that 33 patients (43.4%) had essentially resolved perfusion abnormality areas, 30 (39.5%) had reduced perfusion abnormality ranges compared to preoperatively, 9 (11.8%) had no significant changes, and 4 (5.3%) had increased perfusion abnormality ranges compared to preoperatively. Notably, residual perfusion abnormalities in the successfully recanalized group (32/69, 46.4%) were most frequently observed in the distal MCA branches (18/32, 56.2%) and lenticulostriate arteries (10/32, 31.3%), even after successful recanalization of the proximal occlusive site. Post-thrombectomy, rCBF and rCBV values significantly increased, while rMTT, rTTP, and rTmax values significantly decreased compared to preoperative values (all *P* < 0.05; see Table 2).

**Table 2:** Comparison of relative cerebral perfusion parameters before and after mechanical thrombectomy in 76 AIS patients.

**Parameter**	**Pre-operation**	**Post-operation**	***t*/χ^2^/*Z***	***P*-value**
rCBF	0.56 ± 0.11	1.02 ± 0.29	−11.362	< 0.01
rCBV	0.85 ± 0.13	1.01 ± 0.15	−9.032	< 0.01
rMTT	1.41 (1.34, 1.55)	1.03 (0.91, 1.17)	−6.974	< 0.01
rTTP	1.38 ± 0.12	1.05 ± 0.12	16.321	< 0.01
rTmax	3.7 ± 1.1	1.4 ± 0.9	14.694	< 0.01

AIS, acute ischemic stroke; rCBF, relative cerebral blood flow; rCBV, relative cerebral blood volume; rMTT, relative mean transit time; rTTP, relative time to peak; rTmax, relative time to maximum of the residual function.

Among the 69 successfully recanalized patients, post-CTP scans indicated that 33 (47.8%) had essentially resolved perfusion abnormality areas, 32 (46.4%) still had perfusion abnormality areas (with 28 showing reduced ranges, 4 showing no significant changes, and 1 showing an increase compared to preoperatively); 15 (21.7%) developed hyperperfusion post-thrombectomy, characterized by significantly higher CBF and CBV on the affected side with lower MTT, TTP, and Tmax compared to the healthy side. Hyperperfusion was most common in the MCA cortical territories (12/15, 80.0%), particularly in regions with severe pre-procedural hypoperfusion (rCBF < 0.4).

For the 5 patients who did not achieve successful recanalization, post-CTP scans still showed perfusion abnormality areas, with 2 showing reduced ranges, 2 showing no significant changes, and 1 showing an increase compared to preoperatively. In the non-recanalized group, persistent perfusion deficits were confined to the original occluded territories, with 3/5 cases (60.0%) demonstrating expanded perfusion abnormalities in the distal watershed zones, likely due to progressive ischemia. Notably, 2/5 non-recanalized patients with ICA occlusion showed concurrent hypoperfusion in the anterior cerebral artery territory, suggesting collateral failure.

### 3.4 Changes in perfusion

Abnormality Range Post-Thrombectomy and Clinical Outcomes Among the 76 patients, the post-thrombectomy groups were as follows: 10 (13.2%) with no improvement in perfusion abnormality range, 30 (39.5%) with reduced perfusion abnormality range, and 36 (47.4%) with resolved perfusion abnormality range.

Follow-up mRS scores at 90 days post-surgery showed that 44 patients (57.9%) had good outcomes, while 32 (42.1%) had poor outcomes. Among the 69 patients with successful recanalization, 40 (57.9%) had good outcomes, and 29 (42.1%) had poor outcomes. Comparisons of 90-day postoperative mRS scores among the three groups are presented in Table 2. Patients in the group with resolved perfusion abnormality range post-thrombectomy had significantly higher proportions of improved NIHSS scores at 7 days post-surgery or at discharge compared to the groups with no improvement or reduced perfusion abnormality range (*P*-values of 0.001 and 0.011, respectively). The proportion of patients with good outcomes at 90 days post-thrombectomy was significantly higher in the group with resolved perfusion abnormality range compared to the other groups (both *P* < 0.01; see Table 3 and Figure 1).

**Table 3:** Comparison of general information and clinical outcomes of patients with no improvement, reduced, and resolved perfusion abnormality range post-thrombectomy.

**Category**	**No improvement (10 cases)**	**Reduction (30 cases)**	**Resolution (36 cases)**	***t*/χ^2^/‘**	***P*-value**
Age	62 ± 15	66 ± 13	65 ± 10	0.395	0.675
Male	7	12	23	8.362	0.015
Baseline NIHSS score	14.0 (7.8, 17.5)	13.5 (11.0, 20.0)	12.0 (9.0, 18.5)	0.894	0.640
Admission to hospital from the last normal time	7.0 (2.0, 9.5)	4.5 (1.3, 11.3)	6.0 (3.5, 10.0)	0.892	0.661
Vascular occlusion site	–	–	–	0.000	1.000
ICA	3	8	10		
MCA M1	6	19	21		
MCA M2	1	3	5		
ASPECTS [M (P25, P75), ml]	8.5 (7.8, 10.0)	8.0 (6.0, 9.0)	8.0 (7.5, 9.0)	4.021	0.134
Preoperative infarct core volume	16.0 (3.9, 44.8)	15.1 (4.7, 46.3)	4.0 (0.5, 9.7)	8.594	0.014
7-day or discharge NIHSS improvement, *n* (%)	3 (30.0)	17 (56.7)	26 (72.2)	13.707	< 0.001
90-day mRS score, *n* (%)				26.341	< 0.001
−0–2 分 (good outcome)	2 (20.0)	10 (33.3)	27 (75.0)	–	–

Among the 15 patients who developed hyperperfusion post-thrombectomy, 10 showed improvement in NIHSS scores at 7 days post-surgery or at discharge; 11 had mRS scores of 0–2 and 4 had mRS scores of 3–6 at 90 days post-surgery, with 2 patients dying during hospitalization.

### 3.5 Case presentation

**Figure 2:**
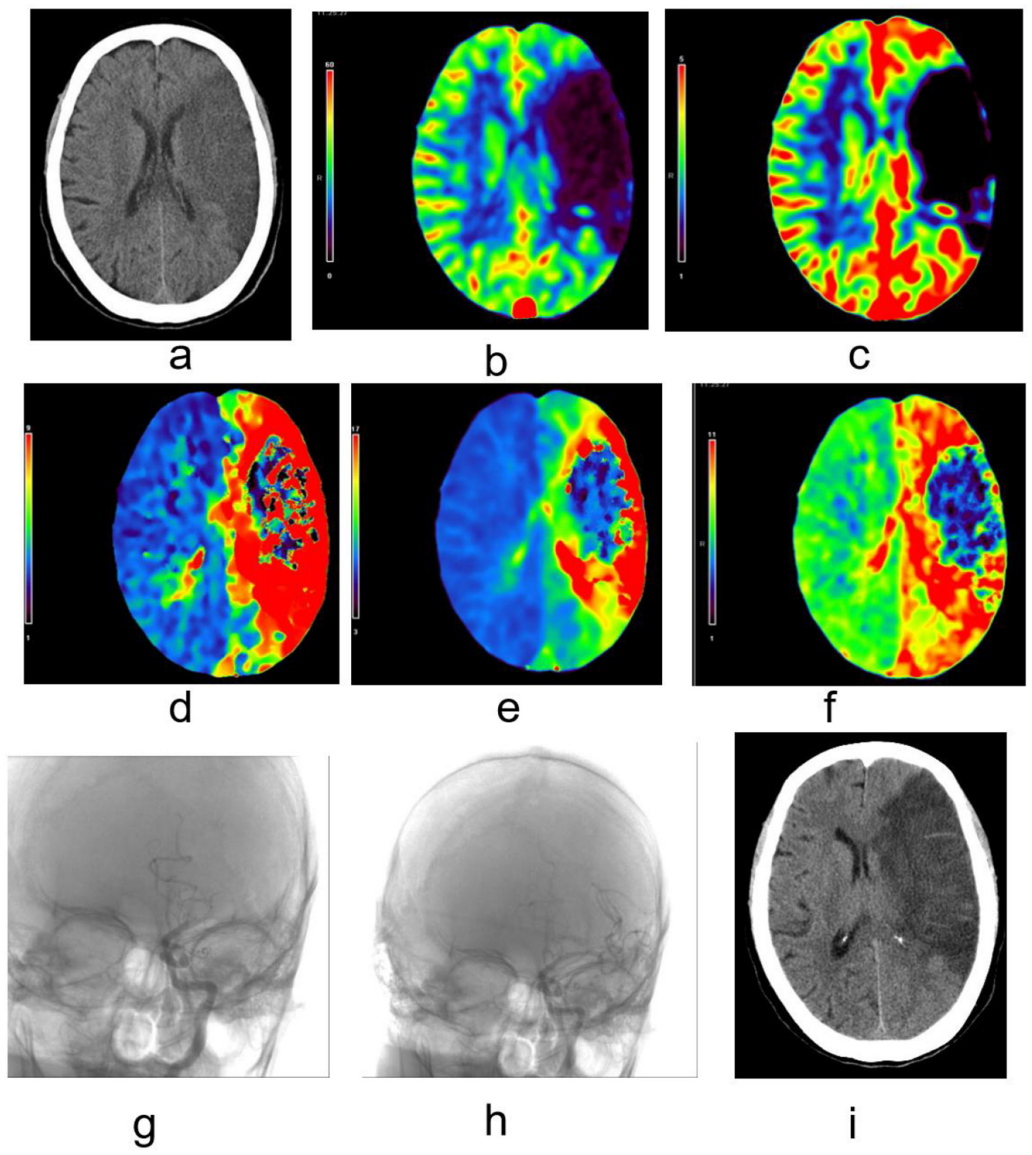
Typical case images; **(a)** Low-density infarct in the left frontal, temporal, and parietal lobes, as well as the left basal ganglia and centrum semiovale; **(b)** Reduced cerebral blood flow (CBF) in the distribution of the left middle cerebral artery; **(c)** Reduced cerebral blood volume (CBV) in the distribution of the left middle cerebral artery; **(d)** Prolonged mean transit time (MTT) in the distribution of the left middle cerebral artery; **(e)** Prolonged time to peak (TTP) in the distribution of the left middle cerebral artery; **(f)** Prolonged delay in the distribution of the left middle cerebral artery, indicating perfusion deficits; **(g)** Pre-thrombectomy DSA confirming occlusion in the M1 segment of the left middle cerebral artery; **(h)** Post-thrombectomy DSA showing mTICI level 3 reperfusion, indicating successful restoration of blood flow in the left middle cerebral artery; **(i)** Post-surgery non-contrast CT showing a decrease in infarct density with more distinct margins, indicating improvement in tissue perfusion and reduction in ischemic damage.

An 85-year-old female presented to our department with a sudden onset of speech difficulties and weakness in the right extremities, 18 h prior to her admission on November 2, 2021, at 17:54. Upon admission, she underwent a CT angiography (CTA) and CT perfusion (CTP) scan, which revealed a low-density infarct in the left frontal, temporal, and parietal lobes, as well as the left basal ganglia and centrum semiovale (Figure 2a). The CTP scan demonstrated reduced cerebral blood flow (CBF, Figure 2b) and cerebral blood volume (CBV, Figure 2c) in the distribution of the left middle cerebral artery, with prolonged mean transit time (MTT, Figure 2d), time to peak (TTP, Figure 2e), and delay (Figure 2f), indicating perfusion deficits.

On November 3, 2021, the patient underwent endovascular thrombectomy and stent-assisted recanalization. The pre-thrombectomy digital subtraction angiography (DSA) confirmed an occlusion in the M1 segment of the left middle cerebral artery (Figure 2g). Following thrombectomy and stent placement, the DSA showed a mTICI level 3 reperfusion, signifying successful restoration of blood flow in the left middle cerebral artery (Figure 2h). A follow-up non-contrast head CT scan performed 46 h post-surgery on November 5, 2021, demonstrated a decrease in the density of the infarct lesion with more distinct margins in the previously mentioned regions (Figure 2i), suggesting an improvement in tissue perfusion and a reduction in ischemic damage following the intervention.

## 4 Discussion

In recent years, numerous randomized controlled trials have confirmed the efficacy of mechanical thrombectomy in achieving favorable clinical outcomes for patients with acute ischemic stroke (AIS) due to large vessel occlusions in the anterior circulation (17, 18). As a result, mechanical thrombectomy has been incorporated as a first-line treatment in international stroke guidelines (19). The mTICI (modified Thrombolysis in Cerebral Infarction) scale is commonly used to evaluate the status of vascular recanalization post-thrombectomy, with mTICI grades 2b to 3 defining successful recanalization, which implies reperfusion of more than 50% of the ischemic area (20). Previous studies have indicated that 80% to 90% of AIS patients with large vessel occlusions can achieve successful recanalization following mechanical thrombectomy (21). In our series of 76 patients, 69 (90.8%) achieved successful recanalization, which is consistent with previous findings.

CT perfusion (CTP) imaging allows for rapid assessment of cerebral hemodynamics and can monitor changes in hemodynamic parameters before and after mechanical thrombectomy, providing a basis for evaluating the efficacy of the procedure. In our study, post-thrombectomy CTP scans showed significant increases in rCBF and rCBV, and decreases in rMTT, rTTP, and rTmax compared to preoperative values (all *P* < 0.05), indicating that mechanical thrombectomy can improve blood perfusion in ischemic brain tissue to some extent. However, despite successful recanalization (mTICI grades 2b to 3), ~50% of AIS patients still have poor outcomes, as measured by an mRS score >2 at 90 days post-surgery (22). In our study, 40.3% of patients with successful recanalization had poor outcomes, which aligns with previous research.

Rubiera et al. (23) found that 63.0% (29/46) of patients with mTICI grade 2b and 42.5% (40/94) with mTICI grade 3 still had perfusion defects on post-thrombectomy CTP. In our cohort of 69 successfully recanalized patients, further stratification by mTICI subgrades (2b vs. 2c−3) revealed distinct perfusion recovery patterns: among 25 patients with mTICI 2b, 18 (72.0%) retained perfusion abnormalities, whereas only 17 (38.6%) of 44 patients with mTICI 2c−3 showed residual perfusion defects (χ^2^=8.92, *P* = 0.003). Correspondingly, complete resolution of perfusion defects was achieved in 7 (28.0%) mTICI 2b patients vs. 27 (61.4%) mTICI 2c−3 patients (χ^2^=9.15, *P* = 0.002), aligning with the graded association between reperfusion extent and tissue-level recovery observed in our perfusion abnormality range analysis. In our 69 successfully recanalized patients, 34 (49.3%) showed near-complete resolution of perfusion defects on post-thrombectomy CTP, while 35 (50.7%) still exhibited perfusion abnormalities, similar to previous findings. Current research suggests that this phenomenon may be related to the no-reflow phenomenon and arterial re-occlusion in the ischemic region (24, 25). The higher residual perfusion defect rate in mTICI 2b cases likely reflects incomplete microvascular reperfusion, even with partial macrovascular recanalization, which is consistent with our observation that mTICI 2b patients had a lower rate of perfusion abnormality resolution (28.0% vs. 61.4% in mTICI 2c−3). In contrast, mTICI 2c−3, characterized by more extensive flow restoration, correlates with better microcirculatory perfusion, as evidenced by our data showing that mTICI 2c−3 patients had a higher proportion in the perfusion abnormality resolved group (27/36, 75.0%) compared to mTICI 2b patients (7/36, 19.4%). Ames et al. (26) first observed the no-reflow phenomenon in a rabbit model of cerebral ischemia in 1968, where microcirculatory reperfusion failed despite the removal of arterial occlusion. Recent studies have shown that AIS patients with successful recanalization post-thrombectomy also experience the no-reflow phenomenon, with factors such as reduced reactivity of capillaries following ischemia and hypoxia, widespread inflammatory responses, and swelling of endothelial cells and astrocytic end-feet potentially causing microcirculatory reperfusion failure. Studies have confirmed that reperfusion is a more accurate predictor of clinical outcomes than recanalization alone, which is supported by our 90-day outcome data: among patients with resolved perfusion abnormalities, 30/36 (83.3%) achieved good outcomes (mRS 0–2), with mTICI 2c−3 patients accounting for 27/30 (90.0%) of these cases. In contrast, mTICI 2b patients had a lower good outcome rate (8/25, 32.0%) even within the successful recanalization cohort, underscoring the clinical significance of distinguishing mTICI 2b from 2c−3 subgrades in predicting functional recovery, as reflected in our perfusion and prognostic analyses.

It is noteworthy that the Chinese population has a higher prevalence of intracranial atherosclerosis (ICAS), which is a well-recognized risk factor for arterial re-occlusion post-mechanical thrombectomy (27, 28). In our cohort, 5 cases (7.5%) experienced re-occlusion during follow-up, with 4 of these cases (80.0%) showing radiological evidence of underlying ICAS on pre-procedural CTA. ICAS-related stenosis may predispose to recurrent occlusion due to progressive luminal narrowing or thrombus formation at the diseased segment, potentially contributing to the residual perfusion abnormalities observed in some patients with successful recanalization (mTICI 2b−3). Compared to populations with lower ICAS burden, the relatively higher re-occlusion rate in our study may partially explain why even among patients achieving mTICI 2c−3, a small proportion (17/44, 38.6%) retained perfusion defects—persistent hemodynamic compromise due to ICAS could hinder complete tissue reperfusion despite macrovascular recanalization. This underscores the need for tailored post-procedural management (e.g., antiplatelet strategies) in ICAS-dominant populations to mitigate re-occlusion risk and optimize perfusion recovery.

In our series of 69 successfully recanalized patients, 14 (20.3%) developed hyperperfusion post-thrombectomy, as indicated by significantly higher CBF and CBV on the affected side with lower MTT, TTP, and Tmax compared to the healthy side. Rubiera et al. (23) reported that 21.2% (32/151) of AIS patients with mTICI grades 2a to 3 experienced hyperperfusion post-thrombectomy on CTP. Ames et al. (26) observed hyperperfusion in 48% (13/27) of AIS patients with successful recanalization (mTICI grades 2b−3) on MRI arterial spin labeling images. The mechanism behind post-thrombectomy hyperperfusion remains unclear and may be associated with the loss of autoregulatory function in the ischemic region's vasculature (29).

Rubiera et al. (23) demonstrated that a Tmax > 6 s region volume of < 3.5 ml on post-thrombectomy CTP is an independent predictor of good outcomes at 3 months post-surgery (OR: 3.5, 95% CI: 1.6 to 7.8, *P* < 0.01). A study (30) retrospectively analyzed pre- and post-thrombectomy CTP images of 82 AIS patients with anterior circulation large vessel occlusions and defined optimal tissue reperfusion as a greater than 90% reduction in the Tmax > 6 s region volume post-thrombectomy compared to pre-thrombectomy, which correlated with good clinical outcomes at 90 days post-surgery (mRS score 0–2) (OR: 8.764, 95% CI: 1.226–62.668, *P*: 0.031). In our study, the proportion of patients with improved NIHSS scores at 7 days post-thrombectomy or at discharge and the proportion with good outcomes at 90 days post-thrombectomy were significantly higher in the group with resolved perfusion defects compared to the groups with no improvement or reduced perfusion defects, suggesting that patients with resolved perfusion defects post-thrombectomy have better clinical outcomes, consistent with previous research. However, our statistical analysis showed that the group with resolved perfusion defects had a smaller infarct core volume preoperatively, and previous studies have also found that patients with optimal tissue reperfusion post-thrombectomy had a smaller infarct core volume preoperatively than those without optimal reperfusion. The relationship between preoperative infarct core volume and post-thrombectomy reperfusion warrants further investigation. In our study, 2 out of 29 patients (6.9%) with resolved perfusion defects post-thrombectomy had poor outcomes, which may be related to ischemia-reperfusion injury.

The clinical utility of post-mechanical thrombectomy (MT) CT perfusion (CTP) is substantial and multifaceted, aligning with observations from our study and relevant literature (23). Post-MT CTP acts as an immediate surrogate of procedural success, as resolution of perfusion abnormalities correlates strongly with favorable 90-day modified Rankin Scale (mRS) scores, offering a more precise assessment of tissue-level reperfusion than angiographic recanalization alone. It enhances prognostication by identifying patients with residual ischemia, who are at higher risk of poor outcomes, and guides rehabilitation planning by stratifying those needing intensive intervention vs. those likely to recover favorably. Regarding hyperperfusion, our finding that 21.7% of successfully recanalized patients developed this phenomenon, with 73.3% achieving good outcomes but 2 experiencing mortality, mirrors broader insights into its dual role as a marker of effective reperfusion and a risk factor for complications. In terms of cost-benefit, post-MT CTP provides incremental value by quantifying perfusion recovery at relatively low additional cost, supporting its potential integration into routine protocols to optimize post-procedural management and resource allocation.

International stroke guidelines recommend pre-treatment CTP assessment in AIS patients to select those suitable for mechanical thrombectomy, while post-thrombectomy CTP has not been widely adopted in clinical practice. Previous studies have often used the change in the volume of perfusion defects (Tmax > 6 s region) before and after AIS treatment to assess the recovery of cerebral blood flow following mechanical thrombectomy. Our study quantitatively evaluated the changes in various CTP-derived hemodynamic parameters before and after mechanical thrombectomy, providing a multifaceted assessment of changes in cerebral perfusion.

This study has several limitations that warrant consideration. First, the retrospective design inherently introduces selection bias, as patients were enrolled based on the availability of pre- and post-thrombectomy CTP data, potentially excluding those with incomplete imaging follow-up and limiting the generalizability of findings. Second, despite comprehensive data collection, some relevant parameters were not fully captured: collateral circulation assessment relied primarily on MP-CTA scores without quantitative analysis of collateral flow volume, and hemorrhagic transformation rates were not systematically stratified by mTICI subgrades, which may obscure associations between reperfusion quality and bleeding risk. Additionally, stroke volume—a critical endpoint reflecting treatment impact on tissue salvage—was not consistently measured in all patients, limiting our ability to directly correlate post-MT perfusion changes with final infarct growth. These gaps highlight the need for prospective studies with standardized acquisition of stroke volume, collateral quantification, and hemorrhagic transformation data to validate our conclusions.

## 5 Conclusion

In summary, the application of CTP allows for the monitoring of cerebral hemodynamic changes in AIS patients following mechanical thrombectomy, offering radiological evidence for the assessment of post-treatment recovery of cerebral perfusion. Clinically, post-thrombectomy CTP holds substantial implications: it enables objective evaluation of tissue-level reperfusion beyond angiographic recanalization, identifies high-risk subgroups such as those with residual ischemia or hyperperfusion for timely intervention, and aids in prognostication to guide rehabilitation planning—aligning with findings that perfusion resolution correlates with improved 90-day mRS outcomes. For future studies, we recommend prospective designs to validate CTP-derived parameters as surrogate endpoints for functional recovery; explore the utility of dynamic CTP in predicting hemorrhagic transformation; and investigate cost-effectiveness in integrating post-thrombectomy CTP into routine protocols, particularly for stratifying patients requiring intensified monitoring or adjuvant therapies.

## Data Availability

The raw data supporting the conclusions of this article will be made available by the authors, without undue reservation.

## References

[1] Zhang Z Pu Y Mi D Liu L. Cerebral hemodynamic evaluation after cerebral recanalization therapy for acute ischemic stroke. Front Neurol. (2019) 10:719. 10.3389/fneur.2019.00719 31333570 PMC6618680

[2] Puntonet J Richard ME Edjlali M Ben Hassen W Legrand L Benzakoun J. Imaging findings after mechanical thrombectomy in acute ischemic stroke: clinical implications and perspectives. Stroke. (2019) 50:1618–25. 10.1161/STROKEAHA.118.024754 31060439

[3] Konstas AA Wintermark M Lev MH. CT perfusion imaging in acute stroke. Neuroimaging Clin N AM. (2011) 21:215–38. 10.1016/j.nic.2011.01.008 21640296

[4] Sundaram VK Goldstein J Wheelwright D Aggarwal A Pawha PS Doshi A. Automated ASPECTS in acute ischemic stroke: a comparative analysis with CT perfusion. Am J Neuroradiol. (2019) 40:2033–8. 10.3174/ajnr.A6303 31727750 PMC6975365

[5] Karlsson A Jood K Björkman-Burtscher IM Rentzos A. Extended treatment in cerebral ischemia score 2c or 3 as goal of successful endovascular treatment is associated with clinical benefit. J Neuroradiol. (2024) 51:190–5. 10.1016/j.neurad.2023.07.005 37532125

[6] Ghozy S Brinjikji W Cloft HJ Kallmes DF. Correspondence on ‘Transradial vs transfemoral access for diagnostic cerebral angiography: frequency of acute MRI findings in 500 consecutive patients at a single center'. J Neurointerv Surg. (2025) 17:229. 10.1136/jnis-2024-021894 38760165

[7] Brugnara G Herweh C Neuberger U Bo Hansen M Ulfert C Mahmutoglu MA. Dynamics of cerebral perfusion and oxygenation parameters following endovascular treatment of acute ischemic stroke. J NeuroInterv Surg. (2022) 14:017163. 10.1136/neurintsurg-2020-017163 33762405 PMC8785045

[8] Kaschka IN Kloska SP Struffert T Engelhorn T Gölitz P Kurka N. Clinical and radiological outcome after mechanical thrombectomy in acute ischemic stroke: what matters? Neuroradiol J. (2016) 29:99–105. 10.1177/1971400916628170 26932163 PMC4978316

[9] Katyal A Bhaskar S. CTP-guided reperfusion therapy in acute ischemic stroke: a meta-analysis. Acta Neurol Scand. (2021) 143:355–66. 10.1111/ane.13374 33188539

[10] Imran R Mohamed GA Nahab F. Acute reperfusion therapies for acute ischemic stroke. J Clin Med. (2021) 10:3677. 10.3390/jcm10163677 34441973 PMC8396980

[11] Heit JJ Wintermark M. Perfusion computed tomography for the evaluation of acute ischemic stroke: strengths and pitfalls. Stroke. (2016) 47:1153–8. 10.1161/STROKEAHA.116.011873 26965849

[12] Potreck A Mutke MA Weyland CS Pfaff JAR Ringleb PA Mundiyanapurath S. Combined perfusion and permeability imaging reveals different pathophysiologic tissue responses after successful thrombectomy. Transl Stroke Res. (2021) 12:1–9. 10.1007/s12975-020-00885-y 33432454 PMC8421283

[13] Wróbel D Wrona P Homa T Jakobschy K Wrona G Sawczyńska K. Sex alters the effect of perfusion deficits on functional outcome in patients with acute ischemic stroke undergoing mechanical thrombectomy. Cerebrovasc Dis. (2024) 54:1–10. 10.1159/000538633 38631293

[14] Sillanpaa N Saarinen JT Rusanen H Hakomaki J Lahteela A Numminen H. CT perfusion ASPECTS in the evaluation of acute ischemic stroke: thrombolytic therapy perspective. Cerebrovasc Dis Extra. (2011) 1:6–16. 10.1159/000324324 22566978 PMC3343752

[15] Chinese Society of Neurology CSS. Chinese guidelines for diagnosis and treatment of acute ischemic stroke 2018. Chin J Neurol. (2018) 51:666–82. 10.3760/cma.j.issn.1006-7876.2018.09.004

[16] Pexman JH Barber PA Hill MD Sevick RJ Demchuk AM Hudon ME. Use of the Alberta stroke program early CT score (ASPECTS) for assessing CT scans in patients with acute stroke. Am J Neuroradiol. (2001) 22:1534–42. 11559501 PMC7974585

[17] Tsivgoulis G Safouris A Katsanos AH Arthur AS Alexandrov AV. Mechanical thrombectomy for emergent large vessel occlusion: a critical appraisal of recent randomized controlled clinical trials. Brain Behav. (2016) 6:e00418. 10.1002/brb3.418 27110444 PMC4834930

[18] Trifan G Biller J Testai FD. Mechanical thrombectomy vs bridging therapy for anterior circulation large vessel occlusion stroke: systematic review and meta-analysis. Neurology. (2022) 98:e1361–e73. 10.1212/WNL.0000000000200029 35173017

[19] Mbroh J Poli K Tünnerhoff J Gomez-Exposito A Wang Y Bender B. The safety and efficacy of mechanical thrombectomy in posterior vs. anterior emergent large vessel occlusion: a systematic review and meta-analysis. J Stroke Cerebrovasc Dis. (2020) 29:104545. 10.1016/j.jstrokecerebrovasdis.2019.104545 31879134

[20] Fischer U Kaesmacher J Mendes Pereira V Chapot R Siddiqui AH Froehler MT. Direct mechanical thrombectomy versus combined intravenous and mechanical thrombectomy in large-artery anterior circulation stroke: a topical review. Stroke. (2017) 48:2912–8. 10.1161/STROKEAHA.117.017208 28887391

[21] Yang Z Zhang G Wu Q Zhu Y Xu S Shi H. Mechanical thrombectomy in anterior vs. posterior circulation stroke: a systematic review and meta-analysis. Interv Neuroradiol. (2024) 30:307–16. 10.1177/15910199221100796 35549748 PMC11310733

[22] Choi JH Im SH Lee KJ Koo JS Kim BS Shin YS. Comparison of outcomes after mechanical thrombectomy alone or combined with intravenous thrombolysis and mechanical thrombectomy for patients with acute ischemic stroke due to large vessel occlusion. World Neurosurg. (2018) 114:e165–e72. 10.1016/j.wneu.2018.02.126 29510288

[23] Rubiera M Garcia-Tornel A Olivé-Gadea M Campos D Requena M Vert C. Computed tomography perfusion after thrombectomy: an immediate surrogate marker of outcome after recanalization in acute stroke. Stroke. (2020) 51:1736–42. 10.1161/STROKEAHA.120.029212 32404034

[24] Ter Schiphorst A Charron S Hassen WB Provost C Naggara O Benzakoun J. Tissue no-reflow despite full recanalization following thrombectomy for anterior circulation stroke with proximal occlusion: a clinical study. J Cereb Blood Flow Metab. (2021) 41:253–66. 10.1177/0271678X20954929 32960688 PMC8370008

[25] Kaul S Methner C Cao Z Mishra A. Mechanisms of the “No-Reflow” phenomenon after acute myocardial infarction: potential role of pericytes. JACC Basic Transl Sci. (2023) 8:204–20. 10.1016/j.jacbts.2022.06.008 36908667 PMC9998747

[26] Ames A 3rd Wright RL Kowada M Thurston JM G Majno. Cerebral ischemia. II. The no-reflow phenomenon. Am J Pathol. (1968) 52:437–53. 5635861 PMC2013326

[27] de Havenon A Zaidat OO Amin-Hanjani S Nguyen TN Bangad A Abbasi M. Large vessel occlusion stroke due to intracranial atherosclerotic disease: identification, medical and interventional treatment, and outcomes. Stroke. (2023) 54:1695–705. 10.1161/STROKEAHA.122.040008 36938708 PMC10202848

[28] Wong LKS. Global burden of intracranial atherosclerosis. Int J Stroke. (2006) 1:158–9. 10.1111/j.1747-4949.2006.00045.x 18706036

[29] Makkawi S Bukhari JI Salamatullah HK Alkulli OA Alghamdi AE Bogari AM. Endovascular thrombectomy after anterior circulation large vessel ischemic stroke: an updated meta-analysis. Syst Rev. (2024) 13:255. 10.1186/s13643-024-02670-6 39396031 PMC11475204

[30] Elsaid N Mustafa W Saied A. Radiological predictors of hemorrhagic transformation after acute ischemic stroke: an evidence-based analysis. Neuroradiol J. (2020) 33:118–33. 10.1177/1971400919900275 31971093 PMC7140299

